# Case report: anti-IL-6 autoantibodies in a patient with immune dysregulation, polyendocrinopathy, enteropathy, X-linked syndrome

**DOI:** 10.3389/fimmu.2025.1660161

**Published:** 2025-09-04

**Authors:** Tiziana Lorenzini, Lars Malmström, Ola Sabet, Samantha Milanesi, Diana Tintor, Severin Walser, Julius Köppen, Maarja Soomann, Mathias Hauri-Hohl, Seraina Prader, Rainer Doffinger, Jana Pachlopnik Schmid

**Affiliations:** ^1^ Pediatric Immunology, University of Zurich, Zurich and the Children’s Research Center, University Children’s Hospital Zurich, Zurich, Switzerland; ^2^ Children’s Cancer Hospital Egypt 57357 (CCHE 57357), Cairo, Egypt; ^3^ Division of Immunology, University Children’s Hospital Zurich, Zurich, Switzerland; ^4^ Division of Stem Cell Transplantation and the Children’s Research Center, University Children’s Hospital Zurich, Zurich, Switzerland; ^5^ Department of Clinical Biochemistry and Immunology, Cambridge University Hospitals NHS Foundation Trust, Cambridge, United Kingdom

**Keywords:** IPEX (Immune dysregulation, Polyendocrinopathy, Enteropathy, X-linked syndrome), T regulatory cells, CD4+ lymphopenia, hypogammaglobulinemia, Th1-like memory phenotype, anti-IL-6 autoantibodies

## Abstract

We describe an atypical presentation of Immune dysregulation, Polyendocrinopathy, Enteropathy, X-linked syndrome. The patient exhibited food allergies and eczema, along with recurrent and severe infections, but notably lacked the hallmark chronic diarrhea and autoimmune polyendocrinopathy. Whole-exome sequencing revealed the hemizygous *FOXP3* variant c.210+1G>T resulting in a loss of protein expression. Immunophenotyping showed an unusual overlap between immune deficiency and immune dysregulation. The patient had CD4^+^ lymphopenia, with a marked reduction of naïve CD4^+^ T cells, and impaired T cell proliferation to specific antigens. Moreover, he had reduced serum levels of immunoglobulin (Ig) G2, IgA, and IgM, but high IgE levels and eosinophilia. Given these features consistent with a cellular and humoral immune defect predisposing to infections, the patient was treated with immunoglobulin replacement therapy, which was beneficial. We identified an altered immunophenotypic signature shared between T regulatory and T effector cells. This T helper 1-like memory phenotype corresponded to an increased secretion of interferon-γ following *ex vivo* stimulation of peripheral mononuclear cells. A key immunological finding was the presence of likely neutralizing anti-IL-6 autoantibodies which, to the best of our knowledge, have never been reported in patients with IPEX syndrome. Although documented later in the disease course, the latter might explain the Hyper IgE syndrome-like features displayed by the patient, including the allergic manifestations in the absence of hyperactivation of the T helper 2 compartment, as well as the poor inflammatory response during infections. This case extends our knowledge of IPEX syndrome by: i) expanding the spectrum of clinical presentations; ii) revealing a distinct phenotypic signature affecting both T regulatory and T effector cells; iii) suggesting that autoantibodies against cytokines may play a previously underappreciated role in shaping the disease manifestations, not only by driving immune dysregulation and allergy but also by impairing immune defense against infections.

## 1 Introduction

The classical triad of early-onset enteropathy, eczema, and type 1 diabetes has been used to clinically define the Immune dysregulation, Polyendocrinopathy, Enteropathy, X-linked (IPEX) syndrome (1). IPEX syndrome is caused by pathogenic variants in the Forkhead Box P3 (*FOXP3*) gene, encoding for the lineage-specific transcription factor that controls T regulatory (Treg) cell signature (2, 3). Treg cells govern immune tolerance to self, symbiotic commensals and fetal antigens (3), and also play a crucial role in host defense and tissue homeostasis (3). In patients with IPEX syndrome, Treg cell stability and suppressive function are invariably impaired (4). As a result of the Treg functional defect, a constellation of autoimmune manifestations is the hallmark of IPEX syndrome (5). Recently, several atypical presentations have been documented, leading to the broadening of the disease spectrum (5). Late-onset and mild cases have been reported, as well as diseases manifesting with cytopenia, gastritis, nephropathy, arthritis, interstitial lung disease, and severe food allergies (5). A genotype-phenotype correlation has been proven to a certain extent, with complete loss-of-function (LOF) leading to a more severe phenotype than missense variants, and variants located in the DNA-binding Forkhead domain being more deleterious than variants located in other domains (6). However, the same pathogenic variant has been detected in patients with different clinical manifestations (6), suggesting the role of genetic modifiers, such as variants in genes encoding for protein partners of FOXP3, and epigenetic factors in determining the expressivity of IPEX syndrome (6, 7).

Most patients with IPEX syndrome do not have a significant reduction of Treg cells, identified by high surface expression of CD25, lack of CD127 expression and the intracellular expression of FOXP3 (1), although a high variability among patients has been described (1, 3). The percentages of naïve and memory T cells, the proliferative response to mitogens, and IgG and IgM levels have been found to be comparable to healthy donors (HDs) (8–10). Immunological abnormalities that have been reported include leukocytosis, an increased CD4:CD8 ratio, eosinophilia, and high IgA and IgE levels (8–10). Here, we describe a unique case of IPEX syndrome characterized by defects in cellular and humoral immunity causing susceptibility to severe infections. Moreover, we investigate the contribution of immunophenotypic alterations and autoantibodies to the disease.

## 2 Case description

### 2.1 Clinical manifestations

**Figure 1:**
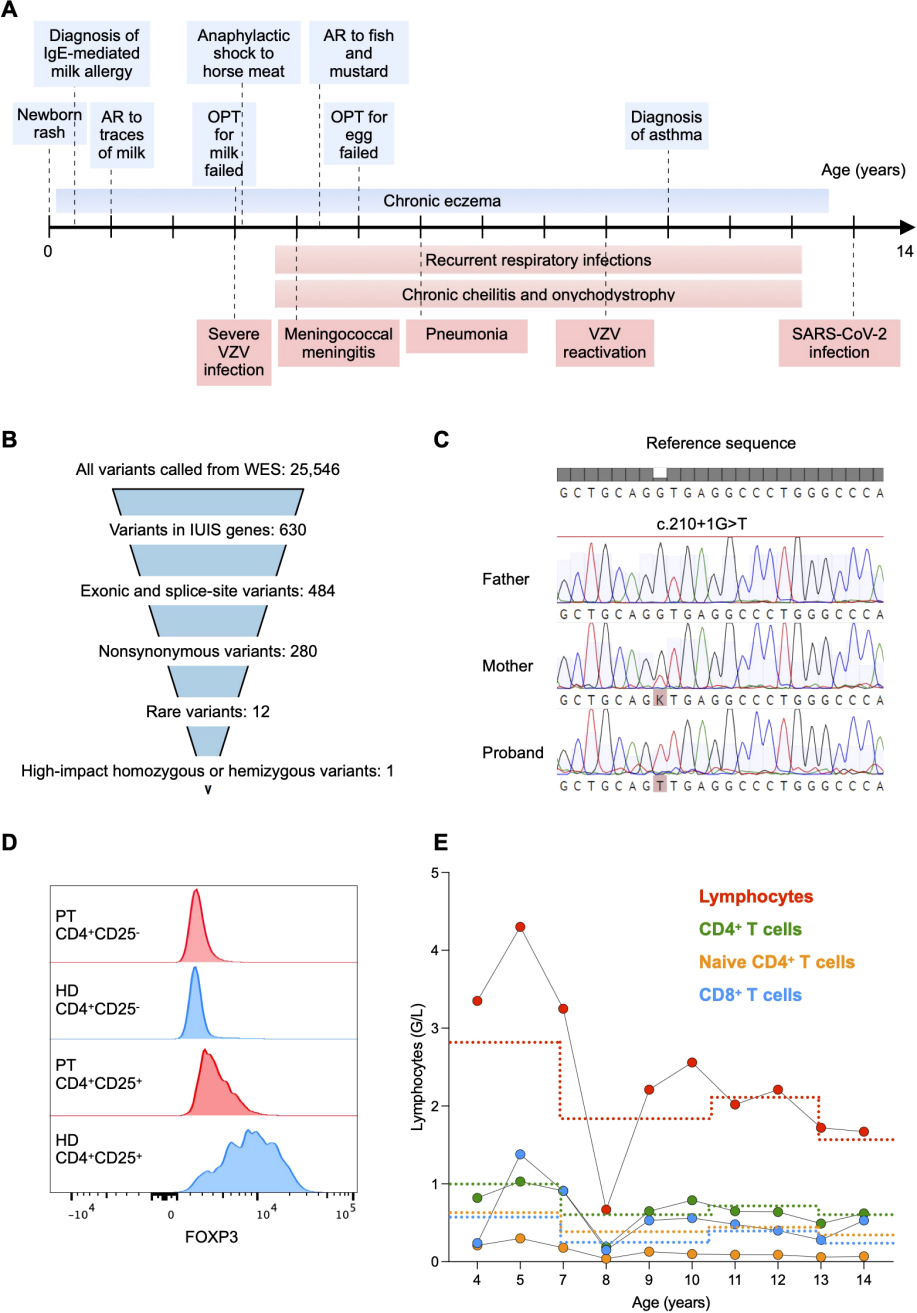
**(A)** Timeline with relevant allergic (light blue) and infectious (light red) manifestations. AR, allergic reaction; OPT, oral provocation test; VZV, varicella-zoster virus. **(B)** Whole exome sequencing (WES) data filtering strategy. We first searched for variants within known causal genes of inborn errors of immunity as reported by the International Union of Immunological Societies (IUIS) (11). **(C)** Sanger sequencing of the *FOXP3* gene showing the X-linked c.210 + 1G>T splice donor site variant in the patient. The variant was absent in the father and monoallelic in the mother. **(D)** Flow cytometry histograms showing FOXP3 expression in CD25^-^CD4^+^ and CD25^+^CD4^+^ T cells, respectively, from the patient (PT) and a healthy donor (HD). **(E)** Total lymphocyte and subtype counts in peripheral blood of the patient. Lower reference values are depicted with a dotted line with the respective color.

The 15-year-old male patient was born to non-consanguineous parents with no familial history of immunological diseases. He initially presented with newborn rash that developed into a persistent eczema which could only be controlled anamnestically by topical antibiotics and was exacerbated by exposure to cow’s milk. From age 1, he demonstrated moderate systemic allergic reactions to cow’s milk, eggs, fish, nuts, and mustard, and anaphylaxis after exposure to horse meat. At age 10, he was diagnosed with asthma. At age 3, he suffered a severe Varicella-zoster virus infection with a bacterial superinfection requiring hospitalization. The lesions resolved very slowly and Herpes zoster was clinically diagnosed at age 8. At age 4, he developed meningococcal meningitis resulting in right-sided hearing loss and partial vestibular impairment despite a timely antibiotic therapy. He also suffered from recurrent respiratory tract infections, including otitis, obstructive bronchitis, and one episode of necrotizing pneumococcal pneumonia at age 5. Interestingly, despite having a lobar pneumonia with pleural effusion and respiratory distress, the patient showed a poor inflammatory response, with low fever and maximal C-reactive protein (CRP) of 39 mg/L (normal range < 10 mg/L). Lung imaging performed at age 12 showed neither bronchiectasis nor interstitial lung disease, although likely post-infectious peripheral perfusion deficits were evident. Pulmonary function, assessed by spirometry, was normal. In addition, he had chronic cheilitis and onychodystrophy, and *Staphylococcus aureus* and *Streptococcus pyogenes* were isolated from cutaneous lesions. At age 12, the patient had a severe acute respiratory syndrome coronavirus 2 (SARS-CoV-2) infection without complications. He did not show manifestations of chronic diarrhea, failure to thrive (**Supplementary Figure 1A**), or autoimmunity. A timeline with the relevant clinical manifestations is shown in **Figure 1A**.

Given the occurrence of eczema, allergies, pneumonia, and susceptibility to staphylococcal infections, the patient was suspected to have a hyper IgE syndrome. However, he did not show skeletal abnormalities, characteristic facial features and high palate, which predict a Signal transducer and activator of transcription 3 (STAT3) deficiency, and his T cell blasts expressed normal levels of Dedicator of Cytokinesis 8 (DOCK8).

### 2.2 Genetic findings

Whole-exome sequencing was performed on the proband and his parents. We first searched for exonic or splice-site non-synonymous rare variants within known causal genes of inborn errors of immunity (11) (**Figure 1B**). We identified the X-linked variant c.210+1G>T (rs886041596) affecting the exon 2 splice donor site within the *FOXP3* gene (NM_014009.4). Sanger sequencing confirmed that the mother was a heterozygous carrier (**Figure 1C**). The variant was predicted to disrupt RNA splicing leading to a LOF (12), was not present in the population databases (GnomAD) (13), and had already been reported in a patient with an IPEX syndrome phenotype (14). We found a low expression of FOXP3 in CD4^+^ T cells using flow cytometry intracellular staining, thus confirming the initial prediction (**Figure 1D**). Therefore, we could classify the variant as pathogenic (Very Strong 1, Strong 1-moderate-, Strong 3, Moderate 2) according to the American College of Medical Genetics and Genomics-Association for Molecular Pathology guidelines (15) and the ClinVar classification (16).

### 2.3 Immunological results

**Figure 2:**
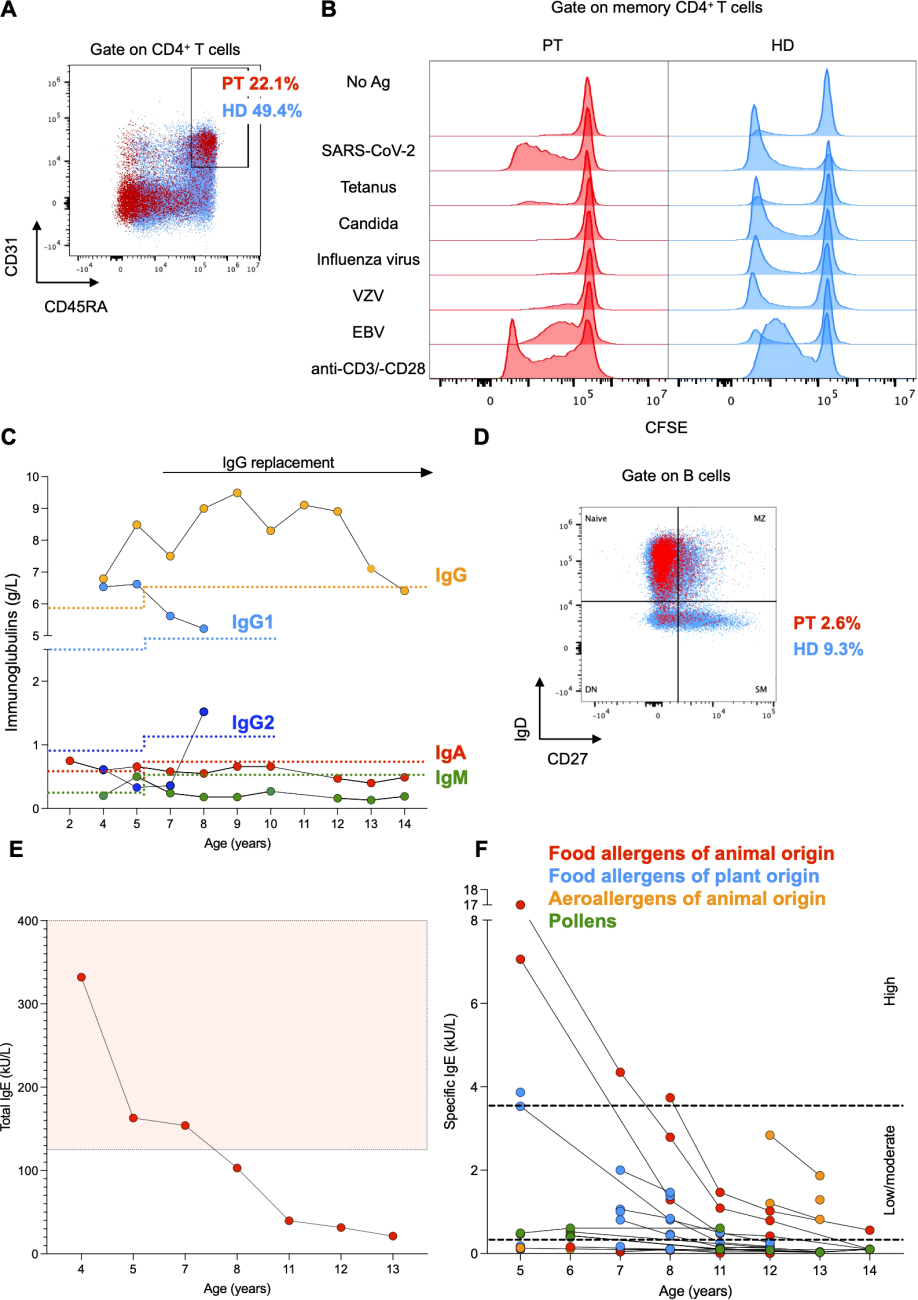
**(A)** Flow cytometry plot of peripheral mononuclear cells from the patient (PT, red) and the mean of 6 age-matched healthy donors (HD, light blue). CD45RA^+^CD31^+^ cells were gated on CD4^+^ T cells. **(B)** Histograms representing proliferation of patient-derived and HD-derived T cells after stimulation with mitogens (anti-CD3 and anti-CD28 beads) and recall antigens. Proliferation was measured as dilution of CFSE on memory CD4^+^ T cells. **(C)** Serum immunoglobulin levels of the patient. Lower reference values are depicted with a dotted line with the respective color. **(D)** Flow cytometry plot of peripheral mononuclear cells from the patient (PT, red) and the mean of 6 age-matched healthy donors (HD, light blue). Switched memory (SM) B cells (CD27^+^IgD^-^) are gated on CD19^+^ **(B)** cells. Naive, marginal zone (MZ) and double negative (DN) B cells are shown. **(E, F)** Total and ImmunoCAP specific serum IgE levels of the patient. Higher total serum IgE values are included within the reference area depicted in light red. Low/moderate and high specific serum IgE values are included within the reference areas delimited by black dotted lines, respectively.

Immunological assessment revealed CD4^+^ T cell counts below the reference range on several occasions, with particularly reduced naïve CD4^+^ T cell counts (**Figure 1E**). The number of CD8+ T cells was within the reference intervals, resulting in a CD4:CD8 ratio of approximately 1:1 (**Figure 1E**). Consistently, the frequency of recent thymic emigrant CD31^+^CD45RA^+^CD4^+^ T cells was decreased as compared to the mean frequency of 6 age-matched HDs, suggesting a reduced thymic output (**Figure 2A**). *In vitro* T cell response to mitogens (anti-CD3/-CD28 beads) and recall antigens was measured by assessing the dilution of carboxyfluorescein diacetate succinimidyl ester (CFSE) as a result of proliferation by flow cytometry. Memory CD4^+^ T cell division in response to polyclonal stimulation with anti-CD3/-CD28 beads was present (**Figure 2B**). SARS-CoV-2-specific cellular immunity was conserved, in line with recent viral exposure, whereas responses to Candida, Influenza virus, and Varicella zoster virus were absent (**Figure 2B**). Given that a previous varicella infection had been documented and the patient was not under immunosuppressive therapy, these findings indicate a partial impairment of CD4^+^ T cell immunological memory. Furthermore, we detected reduced serum levels of IgG2, IgA, and IgM, which could not be explained by renal or enteral losses, but were likely related to the reduced fraction of switched memory B cells (CD27^+^IgD^-^CD19^+^ cells) in comparison to age-matched HDs (**Figures 2C, D**). In light of the impairment of both cellular and humoral immunity, and the history of major bacterial and viral infections, immunoglobulin replacement therapy was started at age 7, resulting in a reduced frequency of infections (**Figures 1A**, **2C**). Total serum IgE and allergen-specific IgE levels (mainly specific for food antigens) were elevated, but tended to decline over time (**Figures 2E, F**). The patient had persistent eosinophilia (**Supplementary Figure 1B**). Parasitic infections were ruled out, and a bone marrow aspirate showed no signs of myeloproliferative or lymphocytic hypereosinophilic syndrome. These findings prompted exploring the role of interleukin-5 (IL-5) as a potential cause of hypereosinophilia (17) and a therapeutic target. However, the serum concentration of IL-5 that was within the reference values at age 14 (0.7 pg/mL, normal values <1 pg/mL) and anti-IL-5 therapy was not considered.

**Figure 3:**
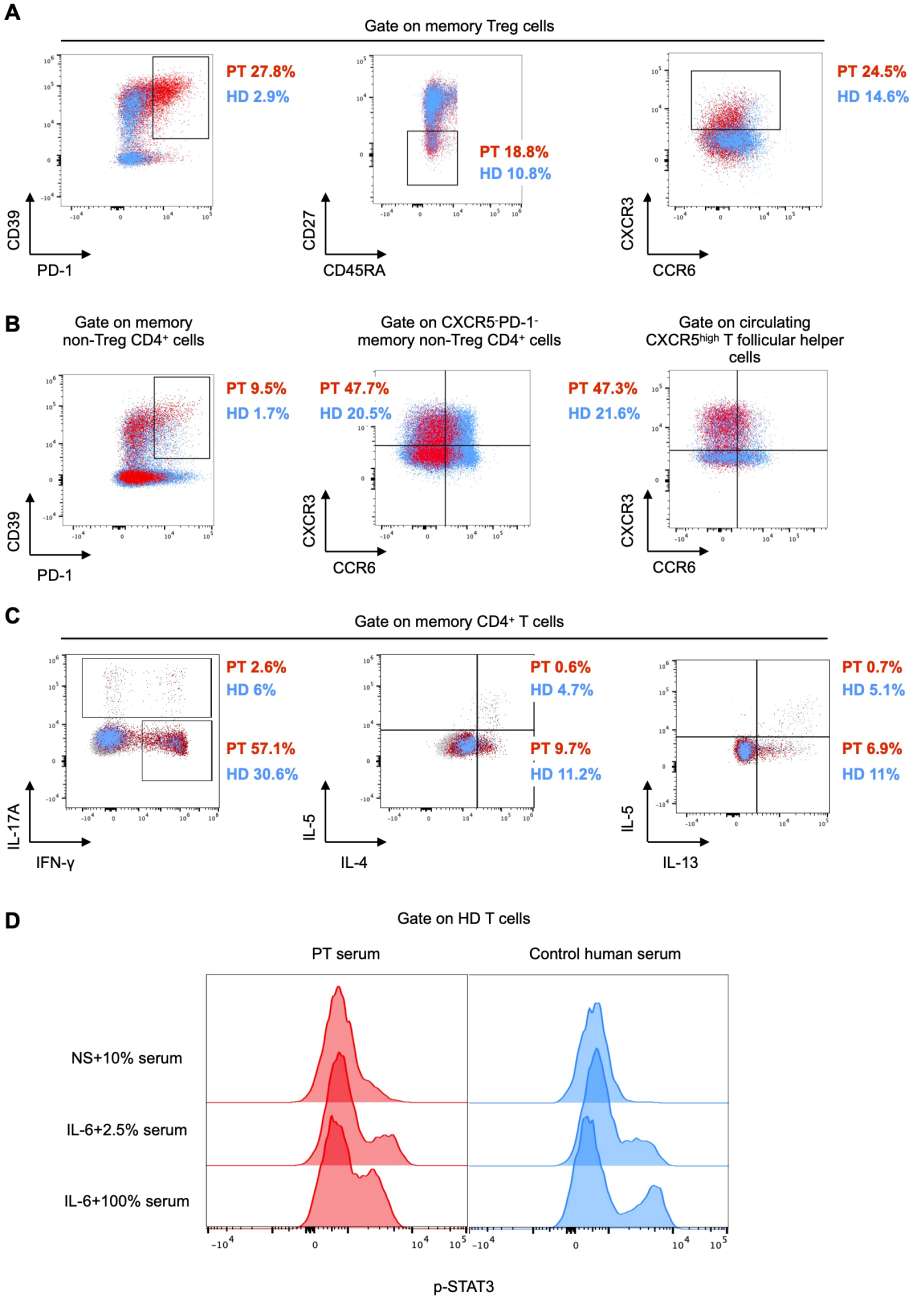
**(A, B)** Flow cytometry plots of peripheral mononuclear cells from the patient (PT, red) and the mean of 6 age-matched healthy donors (HD, light blue). PD-1^+^CD39^+^, CD27^-^, and CXCR3^+^ cells are gated on CD45RA^-^CCR4^+^ (memory) Treg cells. PD-1^+^CD39^+^ are gated on memory non-Treg CD4^+^ cells. CXCR3^+^CCR6^-^ cells are gated on CXCR5^-^PD-1^-^ and circulating CXCR5^high^ T follicular helper cells (defined as CXCR5^high^ memory non- Treg CD4^+^ cells), respectively. **(C)** Flow cytometry plots of peripheral mononuclear cells from the patient (PT, red) as compared to an age-matched healthy donor (HD, light blue). Intracellular IFN-γ, IL-17A, IL-4, IL-5, and IL-13 following stimulation with PMA and ionomycin are gated on memory CD4^+^ cells. **(D)** Flow cytometry histograms showing STAT3 phosphorylation in healthy control peripheral mononuclear cells, either unstimulated (NS) in the presence of 10% patient (PT) or control serum, or stimulated with IL-6 in the presence of 2.5% and 100% patient and control serum, respectively. The expression of phospho-STAT3 is shown in CD3+ cells.

In agreement with previous reports (1, 3), the patient had a normal fraction of CD25^+^CD127^low^ CD4^+^ T cells (**Supplementary Figure 1C**). We investigated the phenotype of this subset by spectral flow cytometry. The proportion of PD-1^+^CD39^+^, CD27^-^, and CXCR3^+^ memory Treg cells was higher in the patient than in HDs (**Figure 3A**). The CXCR3^+^ phenotype has been defined as T helper (Th) 1-like and it is considered to be associated with reduced stability and suppressive function (18). Interestingly, we found elevated frequencies of PD-1^+^CD39^+^ memory non-Treg CD4^+^ cells and a Th1-like phenotype of both memory CXCR5^-^PD-1^-^ T effector (Teff) cells and circulating CXCR5^high^ T follicular helper cells (cTfh) in the patient as compared to HDs (**Figure 3B**).

To investigate whether the Th1-like phenotype observed in both Treg and Teff cells was accompanied by increased secretion of Th1 cytokines, we stimulated peripheral mononuclear cells from the patient and one HD *ex vivo* with phorbol 12-myristate 13-acetate (PMA) and ionomycin in the presence of a protein transport inhibitor. After 5 hours, we assessed cytokine production by intracellular flow cytometry. We measured a higher production of the Th1 cytokine interferon (IFN)-γ in the patient than in the HD, whereas Th2 (IL-5, IL-4, and IL-13), and Th17 (IL-17A) cytokines were not elevated (**Figure 3C**). These results suggested a polarized type 1 response in the patient.

To investigate whether subclinical autoimmunity was present in our patient, we measured several circulating tissue-specific autoantibodies and found them all undetectable, with the exception of anti-glutamate decarboxylase antibodies that were detected at higher levels (33.6 U/mL, normal values < 5 U/mL), although without an apparent pathological correlate (**Supplementary Table 1**). Of note, autoantibodies to the enterocyte antigens harmonin, known to be disease-specific and sensitive (4), were not present, in line with the absence of intestinal manifestations (**Supplementary Table 1**). At age 14, autoantibodies against IFN-α, which have been described in patients with IPEX syndrome (19), were only weakly positive, whereas anti-IL-6 autoantibodies were strongly positive (**Supplementary Table 1**). These autoantibodies persisted for over one year, even following the change in the IgG product used for replacement therapy. We next investigated the functional role of anti-IL-6 autoantibodies by stimulating healthy control T cells with IL-6 in the presence or absence of patient serum. We observed a similar increase in STAT3 phosphorylation upon IL-6 stimulation of T cells cultured with either 2.5% patient serum or control serum (**Figure 3D**). In contrast, only a minimal increase in STAT3 phosphorylation was detected upon IL-6 stimulation in the presence of 100% patient serum, whereas a robust increase was observed with 100% control serum (**Figure 3D**), thus suggesting that the patient serum likely has IL-6 neutralizing properties.

At age 15, the patient was referred for hematopoietic stem cell transplantation as a potential curative treatment for his infection susceptibility and atopy, as well as a potential preventive effect against autoimmune complications, such as diabetes mellitus (20).

## 3 Discussion

The patient described here had an atypical presentation of IPEX syndrome characterized by severe infections, multiple food allergies, and eczema, but without chronic diarrhea and autoimmunity (**Table 1**). The disease presented during the first year of life, but was partially controlled over time. Genetic analysis showed a splice-donor site variant within the N-terminal domain of the *FOXP3* gene, that was shown to disrupt protein expression. Studies in patients and mice have generally shown that variants causing a loss of FOXP3 expression result in a severe phenotype (1, 6). However, studies assessing the effect of different *FOXP3* variants on Treg signatures have demonstrated that LOF variants do not always result in the most profound perturbations (3). Furthermore, genetic defects located outside the *FOXP3* coding regions have been found in patients with atypical phenotypes (5). For example, the splice-site variant c.210+1G>C was described in a patient with late-onset enteropathy, whereas the variant c.210+1G>A was detected in a patient with nephropathy (5). The variant c.210+1G>T, identified in our patient, has been previously reported in a patient with nephropathy, enteropathy, diabetes mellitus and failure to thrive, in the absence of a T cell defect (14). This clinical heterogeneity highlights the complexity of the genotype-phenotype relationship in IPEX syndrome, which may be influenced by genetic modifiers and environmental factors (7).

**Table 1:** Clinical and laboratory features of the patient as compared to a published cohort of patients with IPEX syndrome (10).

IPEX clinical and laboratory features	Patient	IPEX cohort (n=32)
Enteropathy	NO	89.5%
**Eczema**	**YES**	**74.7%**
Alopecia	NO	8.4%
Growth failure	NO	74.7%
Type I diabetes	NO	44.2%
Thyroiditis	NO	16.8%
**Severe infections**	**YES**	**44.0%**
Nephropathy	NO	34.7%
Autoimmune hemolytic anemia	NO	27.4%
Thrombocytopenia	NO	12.6%
Neutropenia	NO	6.3%
Hepatitis	NO	20.0%
**Neurologic**	**YES**	**16.8%**
**Food allergies**	**YES**	**13.7%**
**Pneumopathy**	**YES**	**9.5%**
Lymphoproliferation	NO	9.5%
Arthritis	NO	8.4%
Pancreatitis	NO	2.1%
Cardiac	NO	2.1%
Total white blood cells (G/L) (median)	8.3	11.7
Total lymphocytes (G/L) (median)	2.5	2.4
Total neutrophils (G/L) (median)	3.3	2.2
**Total eosinophils (G/L) (median)**	**1.1**	**1.1**
**Total T CD4+ (G/L) (median)**	**0.7**	**1.6**
Total T CD8+ (G/L) (median)	0.5	0.7
Total NK cells (G/L) (median)	0.7	0.2
Total IgG (g/L) (median)	8.3 (under substitution)	6.6
**Total IgM (g/L) (median)**	**0.2**	**0.53**
**Total IgA (g/L) (median)**	**0.62**	**0.29**
**Total IgE (kU/L) (median)**	**103**	**167**

Pathological manifestations observed in our patient are highlighted in bold.

To the best of our knowledge, this is the first description of CD4^+^ lymphopenia and impaired T cell proliferation to specific antigens in a patient with IPEX syndrome. Although naïve T cell lymphopenia has been described (21), and CD3^+^ lymphopenia has been reported following immunosuppressive therapy (22), this specific CD4^+^ T cell defect has not been previously documented. Several roles of FOXP3 have been outlined that could account for a CD4^+^ T cell defect, thus explaining the observations we made in our patient: i) supporting early thymic maturation to ensure a sufficient output of recent thymic emigrants (23); ii) facilitating post-infectious immune reconstitution to maintain the antigen-specific T cell pool (24); iii) providing protection from premature restimulation-induced cell death (25). While a few patients have been reported to exhibit reduced switched memory B cells and hypogammaglobulinemia, the underlying mechanisms remain unclear (1, 26). We speculate that a defective T cell-dependent class switch recombination might play a role in our patient.

Our patient-derived Treg cells had a unique phenotype characterized by higher PD-1 expression, which has previously been shown in patients with *FOXP3* null variants (27). This feature, together with the increased expression of CXCR3 and downregulation of CD27, has been variably linked to a dysfunctional and unstable Treg cell signature (18, 28, 29).

The notion that pathogenic variants in *FOXP3* have an intrinsic effect on Teff cells is supported by the fact that Teff cells also transiently upregulate FOXP3 upon activation (30). Our results indicate a numerical and functional Teff cell defect, likely contributing to the increased susceptibility to infections. A shared disease signature, including hundreds of genes, has been found in both Treg and Teff cells from patients with IPEX syndrome (3). Consistently, we found that Treg and Teff cells shared similar phenotypical features, such as an effector memory Th1-like profile with increased expression of PD-1. It has been suggested that changes in both Treg and Teff cells are extrinsic and secondary to a systemic response which is unleashed following an initial dysregulation of a few core genes in Treg cells (3).

A shift towards Th1/Tfh1 or excessive IFN-γ production has been reported in patients with chronic infections, autoimmune diseases, and inborn errors of immunity predisposing to autoimmunity (31), as well as in a patient with IPEX syndrome (26). However, more commonly patients with IPEX syndrome display a hyperactivation of the Th2 compartment which is considered to lead to the increased IgE levels and eosinophils (27).

Anti-IL-6 neutralizing antibodies have been previously found in four patients with severe staphylococcal and bacterial infections and no increase in serum CRP (32). A similar phenotype has been observed in patients with autosomal recessive IL-6 receptor deficiency, who also present with clinical features resembling Hyper IgE syndrome, such as atopic dermatitis, high IgE levels, with or without eosinophilia, and normal or reduced class-switched memory B cells (32). Anti–IFN type I, anti–IL-17, and anti–IL-12 antibodies, which have been linked to susceptibility to infections, have been identified in patients with IPEX syndrome (1, 19), although without a proven clinical correlate (19, 33). Other tissue-specific autoantibodies, such as anti-nephrin autoantibodies, have been hypothesized to contribute to atypical manifestations of IPEX syndrome, such as nephropathy (33). Similarly, it is worth considering whether anti-IL-6 autoantibodies, likely impairing the IL-6 response, might account for the atypical phenotype observed in our patient, which mimics conditions associated with impaired IL-6 signaling. These autoantibodies are probably the consequence of a breakdown in peripheral immune tolerance, similar to what is observed in patients with autoimmune polyendocrine syndrome type I, a prototypical defect of central tolerance (33).

However, the fact that the autoantibodies were not measured at the time of the infections is a significant limitation to our conclusions. Despite this limitation, our study highlights intriguing aspects of IPEX syndrome which suggest the value of considering genetic analysis in patients with a Hyper IgE-like presentation.

In conclusion, this case report expands our understanding of IPEX syndrome as heterogeneous disease, with perturbations of both Treg and Teff cells, and complex autoimmune mechanisms.

## Patient’s perspective

From the patient’s perspective, the detection of a pathogenic *FOXP3* variant ended a diagnostic odyssey lasting over a decade and had important implications for his follow-up and therapy. Determining the best treatment for patients with an atypical IPEX syndrome remains challenging. The patient was referred for hematopoietic stem cell transplantation in the setting of good general health, presence of autoantibodies associated with type I diabetes and an available 10/10 matched unrelated donor.

## Data Availability

The raw data supporting the conclusions of this article will be made available by the authors, without undue reservation.
