# Supplementary figures and images for: Case report: anti-IL-6 autoantibodies in a patient with immune dysregulation, polyendocrinopathy, enteropathy, X-linked syndrome
Source: Front Immunol. 2025 Sep 4;16:1660161. doi: 10.3389/fimmu.2025.1660161 (PMC12443674; doi:10.3389/fimmu.2025.1660161)

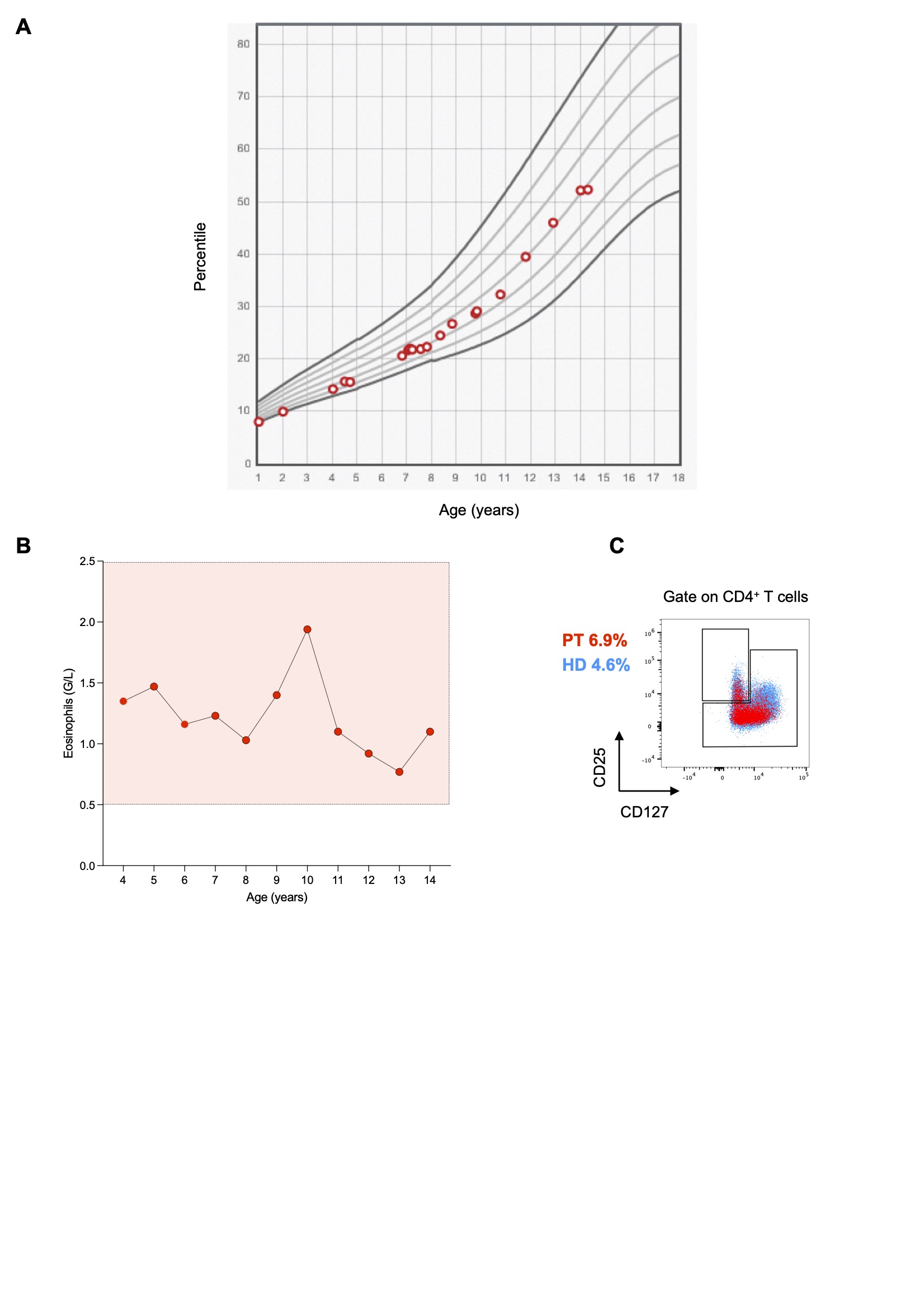

Supplement: Supplementary file 1 [file Image1.jpeg]

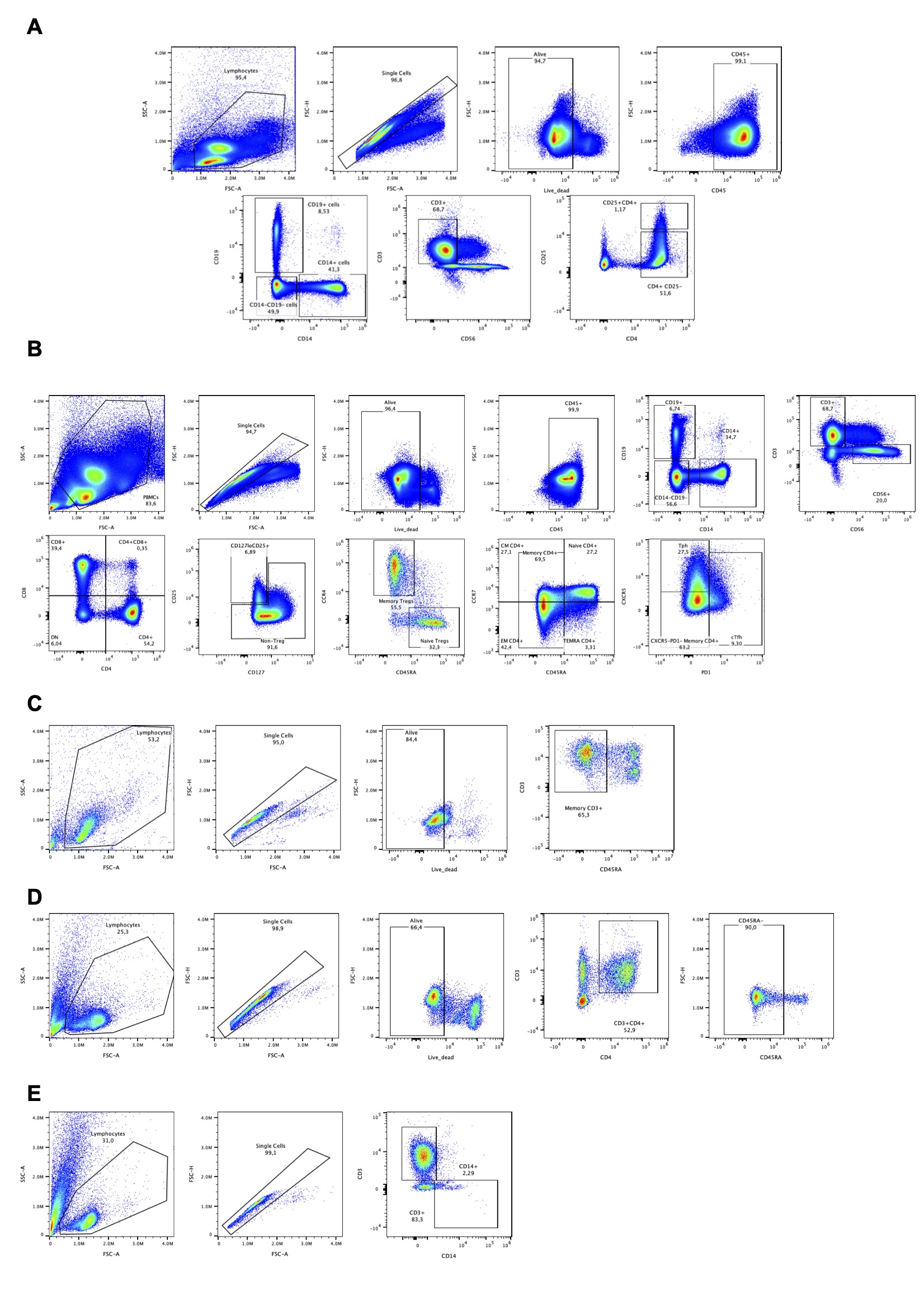

Supplement: Supplementary file 2 [file Image2.jpeg]
