# Supplementary material for: Case report: anti-IL-6 autoantibodies in a patient with immune dysregulation, polyendocrinopathy, enteropathy, X-linked syndrome
Source: Front Immunol. 2025 Sep 4;16:1660161. doi: 10.3389/fimmu.2025.1660161 (PMC12443674; doi:10.3389/fimmu.2025.1660161)
Supplement: Supplementary file 3 [file Table1.docx]

Supplementary Material

# Supplementary Material and methods

**Whole exome sequencing, Sanger sequencing and bioinformatics**

Whole exome sequencing was performed on an Illumina HiSeq2500 system for 2x101 cycles, according to the manufacturer’s instructions (Illumina, San Diego, California). Data pre-processing and identification of the germline short variants were performed using Genome Analysis Toolkit 4 (GATK4, gatk.broadinstitute.org). Functional consequences of each variant were annotated using the Ensembl Variant Effect Predictor (VEP). Sanger sequencing was performed by Microsynth (Microsynth, Balgach, Switzerland) using nested primers: forward GAACCTTCCAGGGCCGAGAT and reverse GACCTTTACTGTGGCACTGGG. The sequencing template was a PCR product amplified with primers: forward ACTCCTTTCCCCTATTGTCTAC and reverse CCTCCTCCTCTCCTGAGAC. PCR amplification was performed under standard conditions using GoTaq G2 Hot Start Green Master Mix (Promega). The resulting amplicon was purified with the QIAquick PCR Purification Kit (Qiagen).

**Flow cytometry**

Peripheral blood mononuclear cells were isolated from blood samples using Ficoll-Paque^TM^ density gradient-centrifugation and cryopreserved. Cells were then thawed and stained with the Zombie Yellow^TM^ Fixable Viable Kit (Biolegend) and the following fluorophore-conjugated anti-human antibodies: anti-CD14 BUV395, anti-IgD BUV563, anti-CD25 BUV661, anti-CD4 BUV737, anti-CD31 BUV805, anti-CD3 Amcyan, anti-CD45RA BV650, anti-CXCR3 PE-CF594, anti-CD38 PE-Cy5, anti-CD56 PE-Cy7 (BD Biosciences), anti-CCR7 BV421, anti-CD127 Pacific Blue, anti-CD45 BV510, anti-CXCR5 BV711, anti-PD-1 BV785, anti-CD27 PerCP-Cy5.5, anti-CCR4 PE, anti-CD39 PE-Fire 810, anti-CD57 AlexaFluor 647, anti-CCR6 Alexa Fluor 700, and anti-CD19 APC-Fire 810 (BioLegend), and anti-CD21 FITC (Beckman Coulter).

To analyze FOXP3 expression, cells were fixed and permeabilised with Foxp3 Transcription Factor Fixation/Permeabilization buffers (eBioscience™). Intracellular staining was performed using V450 anti-FOXP3 antibody (BD Biosciences). For the intracellular cytokine analysis cells were stimulated with 2 µL/mL eBioscience™ Cell Stimulation Cocktail (Thermo Fisher) for 5 hours. Following surface staining, the cells were fixed and permeabilized with the fixation/permeabilization solution kit (BD Biosciences). The following fluorophore-conjugated anti-human antibodies were used for intracellular staining: anti-IFN-γ BV421 (BD Biosciences), anti-IL-5 PE, anti-IL-4 PE/Cy7, anti-IL-13 APC, and anti-IL-17 APC/Cy7 (BioLegend).

Recombinant human IL-6 protein (rhIL-6, PeproTech®) was incubated for 1 hour at 4°C in either 100% patient or control serum, or Gibco Roswell Park Memorial Institute (RPMI) supplemented with 2.5% patient or control serum. Peripheral blood mononuclear cells from a healthy donor were incubated with 10% patient or control serum, or stimulated with 5 ng/mL rhIL-6 for 15 minutes at 37°C. Cells were then fixed using BD Cytofix™ Fixation Buffer (BD Biosciences) and permeabilized with ice-cold 100% methanol. Intracellular staining was performed using the following antibodies: anti-CD14 BUV395 (BD Biosciences), anti-CD3 Alexa Fluor 700 (eBioscience™), and anti-phosho-STAT3 (Tyr705) PE/Cy5 (BioLegend). Samples were acquired using the spectral flow cytometer Cytek Aurora and analyzed using the software FlowJo (FlowJo LLC).

**T cell proliferation**

Cryopreserved peripheral blood mononuclear cells were thawed and labelled at a final concentration of 0.5 µM CFSE (ThermoFisher). Cells were stimulated with anti-CD2/CD3/CD28 beads (Miltenyi) or with the following peptide pools: PepTivator SARS-CoV-2 protein S, M, and N (Miltenyi), Tetanus toxin, Candida (MP65), Influenza A (H1N1) HA, VZV (gE), and EBV EBNA-1 (peptides & elephants) at concentrations according to the manufacturer’s instructions. Proliferation was assessed as CFSE dilution using the spectral flow cytometer Cytek Aurora and the software FlowJo (FlowJo LLC).

# Supplementary Table 1. Circulating autoantibodies measured in the patient with IPEX

|  | 4.8 years | 6.9 years | 14 years |
| --- | --- | --- | --- |
| Antinuclear antibodies | Negative (<1:320) |  | Negative (<1:320) |
| Anti-gliadin deaminated IgG |  | 0 U/mL (<10) |  |
| Anti-gliadin deaminated IgA |  | 2 U/mL (<10) |  |
| Anti-transglutaminase IgA |  | 0 U/mL (<10) |  |
| Anti-tyreoperoxidase |  |  | 5 U/mL (<34) |
| Anti-insulin antibodies |  |  | <0.4 U/mL (<0.4) |
| **Anti-glutamic acid decarboxylase antibodies** |  |  | **38.5 U/mL (<5)** |
| Anti-islet cell antibodies |  |  | <5 U/mL (<15) |
| Anti-zinc transporter 8 antibodies |  |  | <5 U/mL (<15) |
| Autoimmune-Enteropathy related Antigen AIE-75 antibodies |  |  | 9.03 antibody ratio (<10) |
| Anti-IL-17F antibodies |  |  | Negative |
| Anti-IL-17A antibodies |  |  | Negative |
| Anti-IL-22 antibodies |  |  | Negative |
| Anti-IFN-α antibodies |  |  | Weak |
| Anti-IFN-β antibodies |  |  | Negative |
| Anti-IFN-ω antibodies |  |  | Negative |
| Anti-IFN-λ antibodies |  |  | Negative |
| **Anti-IL-6 antibodies** |  |  | **Positive (persisting for over a year)** |

# Supplementary Figures

# 1. (A) Growth chart of the patient with IPEX. Cutoff values corresponding to the 3° and 97° percentile respectively are depicted in dark grey. (B) Eosinophil counts in peripheral blood of the patient. High values are included within the reference area depicted in light red. (C) Flow cytometry plots of peripheral mononuclear cells from the patient (PT, red) and the mean of 6 age-matched healthy donors (HD, light blue). CD25+CD127low cells are gated on CD4+ T cells.

**2.** (A) Flow cytometry gating strategy for Figure 1D. (B) Gating strategy for Figure 2A and D, 3A and B. (C) Gating strategy for Figure 2B. (D) Gating strategy for Figure 3C. (E) Gating strategy for Figure 3D.
